# Relationships of virus titers and transmission rates among sympatric and allopatric virus isolates and thrips vectors support local adaptation

**DOI:** 10.1038/s41598-020-64507-1

**Published:** 2020-05-06

**Authors:** Jessica A. Linak, Alana L. Jacobson, Tim L. Sit, George G. Kennedy

**Affiliations:** 1 Department of Entomology and Plant Pathology, North Carolina State University, Raleigh, NC 27695-7630 USA; 2 Department of Entomology and Plant Pathology, 301 Funchess Hall, Auburn University, Auburn, AL 36849 USA

**Keywords:** Agroecology, Ecological epidemiology, Entomology, Coevolution

## Abstract

Plant viruses rely on insect vectors for transmission among plant hosts, but many of the specifics of virus-vector interactions are not fully understood. *Thrips tabaci*, which transmits *Tomato spotted wilt virus* (TSWV) in a persistent and propagative manner, varies greatly in its ability to transmit different isolates of TSWV. Similarly, TSWV isolates are transmitted at different efficiencies by different populations of *T. tabaci*. This study characterizes differences in virus titers in the vector among TSWV isolate-*T. tabaci* isoline pairings in relation to differences in transmission rates, and demonstrates that although transmission rates were higher for sympatric than allopatric TSWV isolate-*T. tabaci* isoline pairings, virus titers in the thrips vector were significantly lower in the sympatric pairings. Results further demonstrate that TSWV titers in the vector were unrelated to virus titers in the leaf tissue from which they acquired the virus and provide evidence for the importance of specific vector-virus interactions and local adaptation in determining transmission efficiency of TSWV by *T. tabaci*.

## Introduction

Modern agricultural practices and globalization of trade have contributed to a surge of emerging plant viruses that are responsible for billions of dollars in annual crop losses. The expansion of agricultural land alters stable relationships between viruses, insects, and their natural plant hosts; providing opportunities for viruses and vectors to exploit widely available cultivated hosts^1^. Most plant viruses are dependent on insect vectors for plant-to-plant transmission. Specific interactions with their insect vectors are required for the viruses to move to new hosts but little is known about how these interactions impact the efficiency of transmission. This study examines the relationship between virus titer in the vector and transmission efficiency across multiple isolates of *Tomato spotted wilt virus* (TSWV) (Bunyavirales: Tospoviridae) and isolines of its thrips vector, *Thrips tabaci*, originating from multiple geographical locations and host plants. Specifically, we examine differences in transmission rates among TSWV isolate-*T. tabaci* isoline pairings in relationship to differences in virus titers in the vector and whether these relationships differ between sympatric and allopatric pairings of TSWV isolates and *T. tabaci* isolines.

Vector competence reflects the success of the virus in overcoming intrinsic, physiological, microbiota, and immunity barriers, and can be governed by genetic interactions between vector and viral genotypes that are subject to influence by extrinsic factors, including host plants and environmental conditions^2–4^. For persistently transmitted viruses, successful transmission requires that the viruses traverse anatomical barriers in their vectors. After ingestion, the virus must move from the gut lumen, across other tissues or through hemolymph, and into the salivary glands from which the virus can be transferred into plants during insect feeding^5^. Transmission and transmission efficiency of viruses are ultimately determined by the number of virions that accumulate in the appropriate salivary compartments after circulation and/or replication within the vector.

Several studies on vector taxa exhibiting different modes of transmission have shown positive correlations between viral titer within the source plant or insect vector and the efficiency of transmission of plant viruses^6–10^. Circulative, non-propagative viruses in the families Luteoviridae, Geminiviridae, and Nanoviridae are transmitted by aphids, whiteflies or leafhoppers. Because these viruses do not replicate in their vectors, higher virus titers in plants^8–10^ and/or longer feeding periods^11^ have been shown to increase the amount of virus acquired by the vector and increase transmission^12^. In persistent-propagative viruses, less is known about how virus titers in source plants and in the insect vector relate to transmission efficiency. There is evidence in the genus *Tenuivirus* that virus titer in source plants positively correlates with transmission efficiency by planthopper vectors^7^, but this relationship was not consistent among isolates of *Maize stripe virus* (MStV)^6^. Virus titer is also believed to be a determinant for transmission of TSWV because higher titers of TSWV in adult thrips have been related to higher transmission frequencies in the efficient vector *F. occidentalis*; however, differences in transmission among thrips species varying in the efficiency with which they transmit different tospoviruses are not fully explained by virus titers in the vector^13–16^.

Although the aforementioned studies document a relationship between virus titer and transmission frequency for certain virus-vector pairings, they focused on thrips from established laboratory colonies. They do not address the relationship between virus titer in the vector and inter-population variation in transmission within a single vector species or variation among isolates of TSWV. A previous study by Jacobson and Kennedy^17^ examining transmission of 89 distinct pairings between TSWV isolates and *Thrips tabaci* isolines showed a significant effect of virus isolate, thrips isoline, and their interaction on transmission efficiency. Although transmission rates ranged from 0–55% across all isolate by isoline pairings, the ability of a single *T. tabaci-*isoline to transmit multiple virus isolates varied up to 18-fold, and the transmission rates of each TSWV isolate by multiple *T. tabaci* isolines varied up to 45-fold. In addition, significantly higher transmission rates were observed among sympatric (originate from the same location) TSWV isolate-*T. tabaci* isoline pairings than allopatric pairings (originate from different locations), suggesting local adaptation between virus and vector resulting from coevolution in which local virus has greater infectivity than foreign virus on local vectors^18^. The purpose of this study was to examine whether or not the observed variation in transmission frequency was influenced by differences in virus titer within the vector. Both the leaf tissue and thrips used in the studies of Jacobson and Kennedy^17^ were flash frozen in liquid nitrogen immediately following their acquisition and inoculation access periods, respectively, and stored at −80 °C until used in this study. We used a subset of the TSWV isolate and *T. tabaci* isoline pairings studied by Jacobson and Kennedy^17^ to determine if TSWV titers in adult *T. tabaci* varied among the TSWV isolate and *T. tabaci* isoline pairings. We also examined whether differences in transmission rates were related to variation in virus titers in the leaf tissue from which the thrips acquired the virus and in the thrips vector.

## Results

*Thrips tabaci* and TSWV isolates included in our study were subsamples of those tested by Jacobson and Kennedy^17^ in their characterization of differences in transmission efficiency among different pairings of *T. tabaci* isolines and TSWV isolates, both of which were obtained from multiple locations and host plants in North Carolina. The *T. tabaci* individuals studied were from clonal isolines of *T. tabaci* that were established from thelytokous females (parthenogenetic reproduction - producing only female offspring) collected at each location to minimize genetic variation within each of the isolines. Transmission efficiency of each TSWV isolate by each *T. tabaci* isoline was then characterized. Details of collection, establishment of clonal thrips isolines, TSWV isolates, transmission experiments, and results from transmission assays to characterize each TSWV isolate-isoline pairing are described in Jacobson and Kennedy^17^. They classified individual thrips as transmitting or non-transmitting based on a DAS-ELISA test of the leaf discs on which viruliferous thrips were allowed to feed during the inoculation access period (these leaf discs were no longer available for the experiments reported here). Samples of transmitting thrips, non-transmitting thrips, and infected leaf tissue used for acquisition of TSWV from each isolate-isoline pairing were flash frozen in liquid nitrogen and stored at −80 °C until used in the experiments reported here. A subset consisting of 12 of the 89 TSWV isolate-*T. tabaci* isoline pairings for which transmission efficiencies were reported by Jacobson and Kennedy^17^ were chosen for the experiments reported here; they were representative of the range in transmission efficiencies observed among the 89 TSWV isolate-*T. tabaci* isoline pairings and included both sympatric and allopatric pairings^17^ (Table 1). For each pairing, we used RT-qPCR to quantify TSWV titers in individual transmitting and non-transmitting thrips, and in the TSWV-infected, *Emilia sonchifolia* leaf tissue from which the thrips acquired the virus. Up to five transmitting and non-transmitting thrips were selected per TSWV isolate-*T. tabaci* isoline pairing unless transmission rates were so low that five transmitting individuals were not observed in transmission experiments. The numbers of transmitting and non-transmitting thrips for each TSWV isolate-*T. tabaci* isoline pairing subjected to RT-qPCR are shown in Supplementary Table S1.

**Table 1:** TSWV isolate and T*hrips. tabaci* isoline pairings: North Carolina locations and host plants from which TSWV isolates and adult thrips used to initiate each *T. tabaci* isoline were collected and mean proportion of *T. tabaci* transmitting TSWV for each isolate-isoline pairing as reported by Jacobson and Kennedy (2013). * indicates sympatric isolate-isoline pairing.

TSWV isolate	*T. tabaci* isoline	Location isolate-isoline	Host plant Isolate-isoline	Proportion thrips transmitting (n)
AM1	IPOC1*	Cove City – Cove City	*Nicotiana tabacum –Allium spp*.	0.20 (n = 65)
AM1	Kin1	Cove City – Kinston	*N. tabacum – Allium cepa*	0.21 (n = 66)
AM1	SH2	Cove City – Jackson Springs	*N. tabacum - Secale cerealae*	0.21 (n = 28)
AM1	SH72	Cove City – Jackson Springs	*Capsicum annuum – Raphanus sativus var niger*	0.10 (n = 63)
SH3	IPOC1	Jackson Springs - Cove City	*N. tabacum – Allium spp*.	0.03 (n = 67)
SH3	Kin1	Jackson Springs – Kinston	*N. tabacum – A. cepa*	0.16 (n = 68)
SH3	SH2*	Jackson Springs – Jackson Springs	*N. tabacum – S. cerealae*	0.16 (n = 131)
SR3–3	IPOC1*	Cove City – Cove City	*N. tabacum – Allium spp*.	0.07 (n = 54)
SR3-3	Kin1	Cove City – Kinston	*N. tabacum – A. sepa*	0.08 (n = 42)
SR3-3	SH2	Cove City – Jackson Springs	*N. tabacum – S. cerealae*	0.06 (n = 141)
SR3-3	SH72	Cove City – Jackson Springs	*N. tabacum - Raphanus sativus var niger*	0.20 (n = 20)
SHP	SH2*	Jackson Springs – Jackson Springs	*Capsicum annuum - S. cerealae*	0.55 (n = 52)

### Primer efficiency and validation

Most of the published RT-qPCR primers for thrips internal controls were designed for *Frankliniella occidentalis* and did not show the specificity and consistency required for reproducible RT-qPCR with *T. tabaci* samples (Supplementary Table S2). EF1A primers from *F. occidentalis* demonstrated the most promising results for a heterologous internal control. However, homologous *T. tabaci* EF1A primers (Supplementary Table S3) amplified more robustly and consistently (Supplementary Fig. S1). Primer pair EF1A_346F and EF1A_456R was chosen as the internal control with a primer efficiency of 86% (Table 2).

**Table 2:** Primers used in RT-PCR reactions.

Primer Name	Sequence (5′-3′)
EF1A_346F	CGTCAAGGAACTTCGTCGTG
EF1A_456R	CACAGGGGTGTATCCGTTG
TSWVL_4382F	GCATGAAYTGGTTRCAAGGC
TSWVL_4493R	CAGAGTGCACAATCCATCTAG
Emilia_5.8 *S*_rRNAF	GTGTGAATTGCAGAATCCCGT
Emilia_5.8 *S*_rRNAR	CATGTGACGCCCAGGCA

The elongation factor 1 alpha (EF1A) primers were used as a reference sequence in *Thrips tabaci*. TSWVL primers were used to target the L RNA sequence for TSWV, and the Emilia_5.8S primers were based on the reference sequence for *Emilia sonchifolia*.

Because *E. sonchifolia* does not have published RT-qPCR primers or a complete genome sequence, and previously published RT-qPCR primers developed from the highly conserved common plant genes actin, tRNA and profilin^19^ failed to amplify from *E. sonchifolia* source tissue, an alignment of 3 partial *E. sonchifolia* sequences for the 5.8 *S* rRNA gene and internal transcribed spacer (ITS) were utilized to design additional primer pairs (Supplementary Table S4). The ITS primers had poor amplification but the 5.8 *S* rRNA primers amplified most efficiently and consistently (Supplementary Fig. S2). Primer pair Emilia_5.8*S*_rRNAF and Emilia 5.8*S*_rRNAR was chosen as the internal control for the source leaf tissue with a primer efficiency of 97%.

The N gene primers for TSWV quantification produced inconsistent results (Supplementary Table S5). The L RNA primers were more consistent and robust overall than any of the N primers. Primer pair TSWVL4832F and TSWVL 4493 R was used to measure TSWV transcript levels with a primer efficiency of 92%. The L RNA is also a better representation of the viral titer present in infected tissue because it measures mostly genome replication, while the N gene primers measure both genome replication (S RNA) and N gene expression (amplified S mRNAs).

### Viral titers in the source leaf tissues

**Figure 1:**
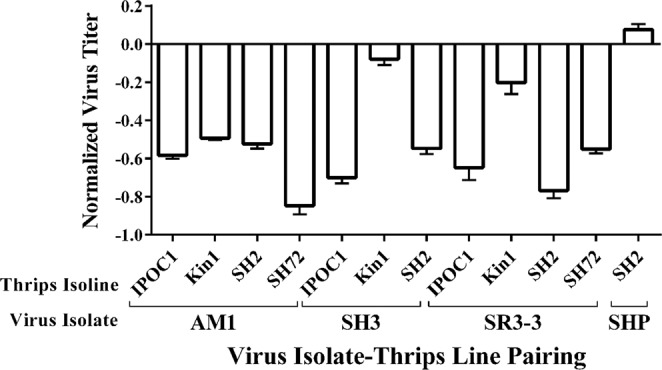
Log mean relative abundance ratio for TSWV LRNA in *Emilia sonchifolia* source leaf tissue for all TSWV isolate-*Thrips tabaci* isoline pairings. No significant differences in titer were found between any of the isolate-isoline pairings at *P* = 0.05. Error bars = standard error of mean.

There were no statistically significant differences in virus titers in the source leaf tissue used for virus acquisition among any of the TSWV isoline and *T. tabaci* isolate pairings [F = 0.25, df = (11, 12), *P* = 0.9853] (Fig. 1; Table 3). This result indicates that titer in the source leaves does not account for differences in virus titer or transmission among thrips included in this study because all thrips had the potential to ingest similar amounts of TSWV. The Ct values of the TSWV L RNA are very similar to the Ct values of the 5.8 *S* rRNA of *E. sonchifolia* showing the virus replicates to the level of ribosomal RNA in the source leaf tissue.

**Table 3:** Average Ct values (+ standard error) for the *Emilia sonchifolia* leaf tissue and *Thrips tabaci* individuals^a^.

TSWV Isolate	Thrips Isoline	*E. sonchifolia* Ct Values		*T. tabaci* Ct Values (sd)	
		TSWV-L RNA	Emilia 5.8*S* rRNA	TSWV-L RNA	Thrips EF1A
AM1	IPOC1*	14.7 ± 0.1	12.2 ± 0.1	30.5 ± 0.5	21.3 ± 0.1
AM1	Kin1	14.5 ± 0.0	12.3 ± 0.1	25.3 ± 0.2	20.8 ± 0.1
AM1	SH2	14.9 ± 0.1	12.5 ± 0.1	29.6 ± 0.2	19.6 ± 0.1
AM1	SH72	16.1 ± 0.3	12.6 ± 0.1	31.2 ± 0.3	18.9 ± 0.2
SH3	IPOC1	14.6 ± 0.2	11.7 ± 0.1	27.3 ± 0.3	20.6 ± 0.1
SH3	Kin1	12.6 ± 0.1	11.9 ± 0.2	34.2 ± 0.2	21.7 ± 0.3
SH3	SH2*	14.4 ± 0.2	12.0 ± 0.1	35.8 ± 0.7	18.1 ± 0.0
SR3-3	IPOC1*	15.2 ± 0.4	12.4 ± 0.1	30.0 ± 0.2	22.7 ± 0.1
SR3-3	Kin1	13.0 ± 0.4	11.8 ± 0.1	31.4 ± 0.2	21.2 ± 0.1
SR3-3	SH2	14.1 ± 0.2	10.9 ± 0.2	27.0 ± 0.2	22.5 ± 0.2
SR3-3	SH72	15.0 ± 0.0	12.5 ± 0.1	27.1 ± 0.3	24.2 ± 0.1
SHP	SH2*	12.7 ± 0.1	12.4 ± 0.1	25.7 ± 0.3	20.9 ± 0.1

^a^Values within columns are not significantly different (P > 0.05).

*TSWV isolate and thrips isoline pairings that are designated as sympatric.

### Effects of TSWV isolate and *T. tabaci* isoline on virus titer in thrips

**Figure 2:**
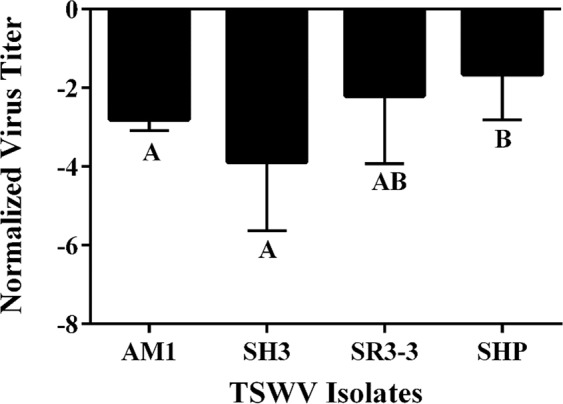
Mean normalized Log_10_ transformed virus titer of each *Tomato spotted wilt virus* (TSWV) isolate quantified from individual thrips. The main effect of virus isolate on titer is significant (*P* = 0.0011). Mean separation of TSWV isolates by LS Means at α = 0.05. Error bars = standard error of mean.

**Figure 3:**
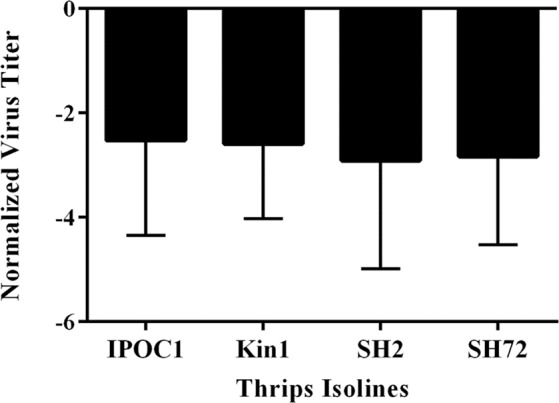
Mean normalized Log_10_ transformed titer of *Tomato spotted wilt virus* (TSWV) isolates averaged by *Thrips tabaci* isoline. The y-axis shows normalized virus titer of all thrips, both non-transmitting and transmitting. TSWV titers were not significantly different among isolines at *P* = 0.05. Error bars = standard error of mean.

**Figure 4:**
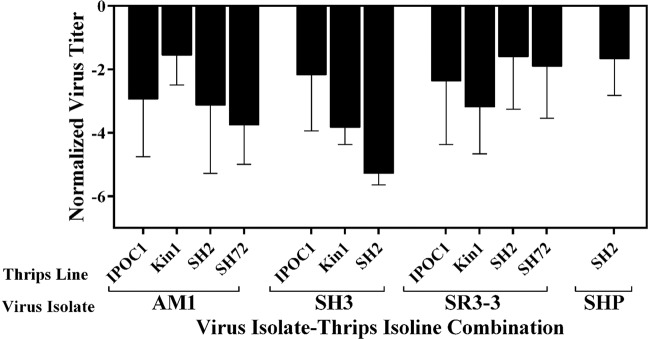
Mean normalized log_10_ transformed virus titer compared across all *Tomato spotted wilt virus* (TSWV) isolate-*Thrips tabaci* isoline pairings. Normalized TSWV titer means for all thrips, including transmitting and non-transmitting, shows titer is dependent on the interactions of the virus isolate and thrips isoline (*P* < 0.001). Mean separation of TSWV isolates by LS Means at α = 0.05. Error bars = standard error of mean.

**Figure 5:**
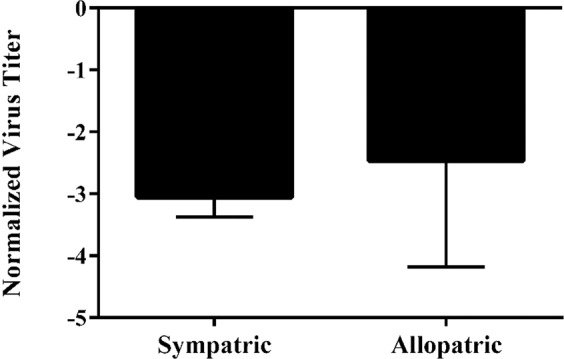
Mean normalized log_10_ transformed *Tomato spotted wilt virus* (TSWV) titer of sympatric and allopatric TSWV isolate-*Thrips tabaci* isoline pairings. The main effect of sympatry on titer is significant (*P* = 0.0002), meaning allopatric pairings have higher titers on average compared to sympatric pairings. Mean separation of TSWV isolates by LS Means at α = 0.05. Error bars = standard error of mean.

The main effect of TSWV isolate on virus titer per thrips was highly significant [F = 8.6, df = (3, 93), *P* < 0.0001] (Fig. 2), whereas the main effect of *T. tabaci* isoline [F = 2.3, df = (3, 93), *P* = 0.0820] (Fig. 3) was not. However, the isolate by isoline interaction was highly significant [F = 6.12, df = (5, 93), *P* < 0.0001 (Fig. 4)], indicating that the effect of TSWV isolate on virus titer per thrips varied depending on the *T. tabaci* isoline. In Jacobson and Kennedy^17^ this significant interaction term was consistent with higher transmission efficiency among sympatric than allopatric isolate-isoline pairings. Therefore, another analysis was conducted in which collection location (allopatric/sympatric) replaced the interaction term in the ANOVA model. Titers were measured in 39 individual thrips representing sympatric TSWV isolate-*T. tabaci* isoline pairings and 66 individual thrips representing allopatric TSWV isolate-*T. tabaci* isoline pairings. In this analysis, virus isolate [F = 12.21, df = (3, 94), *P* < 0.0001] and *T. tabaci* isoline [F = 5.53, df = (3, 94), *P* = 0.0015] main effects on virus titers per thrips were both significant. In addition, the effect of location was significant; mean virus titers of adult thrips were significantly lower for sympatric than allopatric virus isolate-thrips isoline pairings [F = 15.13, df = (1, 97), *P* = 0.0002] (Fig. 5).

### Relationship between TSWV transmission and virus titer in the vector

**Figure 6:**
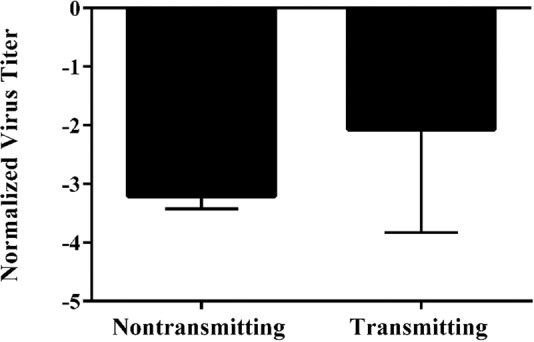
Mean normalized log_10_ transformed *Tomato spotted wilt virus* TSWV titer in transmitting thrips vs non-transmitting thrips. The main effect of transmission on titer is significant (*P* = 0.0011). Mean separation of TSWV isolates by LS Means at α = 0.05. Error bars = standard error of mean.

Of the 105 *T. tabaci* included in our study, 42 percent had transmitted TSWV to leaf discs and 58 percent had not transmitted. TSWV was detected in all 105 thrips regardless of whether they transmitted the virus, and the mean virus titer per thrips was significantly higher in transmitting than non-transmitting thrips [F = 11.23, df = (1, 100), *P* = 0.0011] (Fig. 6). However, even within individual TSWV isolate-*T. tabaci* isoline pairings virus titers were not always higher in transmitting than non-transmitting thrips, demonstrating that non-transmitters could support similar levels of viral replication (Supplementary Table S6; Supplementary Fig. S3). Because we measured virus titers in only a small sub-sample (n = 5–10) of the individual thrips used by Jacobson and Kennedy^17^ to characterize the transmission efficiency of each TSWV isolate-*T. tabaci* isoline pairing included in this study (Table 1), our subsamples could not be used to directly test associations between virus titers and transmission efficiencies of the individual isolate and isoline pairings.

## Discussion

The aims of this study were to characterize the relationship between virus titers in a thrips vector and transmission rates of TSWV, and to test whether these relationships differ between sympatric and allopatric pairings of the virus isolates and vector isolines. During these experiments, we developed a reliable method for quantifying L RNA, which is the only genome segment of TSWV that does not produce subgenomic RNA and, therefore, provides a measure of only genomic replication and not gene expression. In our study, virus titer in the leaf tissue from which thrips acquired virus did not account for differences in virus titers within thrips, suggesting vector-virus interactions are more important determinants of virus titers reached within thrips than viral load acquired during feeding.

Virus titers in adult *T. tabaci* did not fully explain the variation in transmission efficiency observed among the TSWV isolate-*T. tabaci* isoline pairings. Although mean virus titer in transmitting individuals was significantly higher than in non-transmitting individuals, there was extensive variation in virus titers among transmitting and non-transmitting individuals, both within and among TSWV isolate-*T. tabaci* isoline pairings. The effects of TSWV isolate and the interaction of TSWV isolate and *T. tabaci* isoline were consistently significant in all of our analyses, whereas the effect of *T. tabaci* isoline was significant only when collection location (sympatry/allopatry) was included as a main effect. Jacobson and Kennedy^17^, in their prior study involving a larger number of TSWV isolate-*T. tabaci* isoline pairings, found that transmission was higher in sympatric TSWV isolate-*T. tabaci* isoline pairs than allopatric pairs; a result indicative of local adaptation^20^. Within the subset of TSWV isolate-*T. tabaci* isoline pairings that were included in our study, the mean transmission rates for the sympatric and allopatric pairings were 25% and 12%, respectively, but the mean virus titers in thrips from the allopatric pairings were significantly higher than in the thrips from the sympatric pairings. This finding contrasts with our analysis comparing all transmitting and non-transmitting thrips, which showed that mean virus titers were higher in thrips that transmitted than in those that did not, and suggests that local adaptations between virus and vector leading to more efficient virus transmission involves more than simply higher virus replication or accumulation rates in the vector. In the collection locations for *T. tabaci* and TSWV isolates used in our study the most abundant vectors are *F. fusca* and *F. occidentalis*. The evidence for local adaptation between TSWV isolates and *T. tabaci* populations used in our study suggests that selection on the virus population for adaptation to specific vectors is sufficiently strong that even minor vector species such as *T. tabaci* can influence local vector-virus evolution^17,21^ (Table 1).

A genetic basis for differences in transmission efficiency of tospoviruses by *T. tabaci* and two other thrips species has been documented^22–24^ but the underlying mechanisms responsible for the differences have not been determined. Although TSWV titers in the vector *F. occidentalis* have been positively associated with vector competence (transmission yes or no) and frequency of transmission^15,16^, virus titers in the thrips are not necessarily indicative of titers in the salivary gland and salivary reservoir from which the virus is carried in saliva to the plant during feeding. There are numerous opportunities for virus replication and movement within the vector that may be compromised, resulting in reduced transmission efficiency^25,26^. Viral genes that encode nucleocapsid, glycoproteins, and NSs have been implicated in infection and movement in thrips vectors^27^–^31^. For TSWV to be transmitted by thrips, it must traverse the midgut following ingestion by first instars and infect the muscles surrounding the midgut where replication occurs. The virus must then move via the tubular salivary glands and the efferent duct that leads from the principal salivary glands to the salivary reservoir, and the level of salivary gland infection has been shown to be a critical determinant of transmission^32–34^. Viral receptor proteins putatively interact with the receptors of midgut cells in thrips, enabling cell-to-cell movement of TSWV via endocytosis to the salivary glands for transmission^29^,^31^,^35^–^37^. Even if replication occurs, infection may be limited to the midgut if virus cannot cross the basal lamina^38^, and midgut-limited infections can persist in adult thrips that cannot transmit the virus, as well as in non-vector species that have fed on virus infected plant tissue^39,40^. In aphids, the basal lamina of the accessory salivary gland acts as a selective barrier associated with differential transmission of *Barley yellow dwarf virus*^41–43^ and specific cells in the primary salivary gland control differential transmission of *Tomato yellow leaf curl China virus* and *Tomato yellow leaf curl virus* by different whiteflies in the *Bemisia tabaci* cryptic species complex^44^.

TSWV has been shown to elicit an immune response in *F. occidentalis* and *F. fusca*^16,45–47^ that limits virus movement, suggesting variation in vector competence may be affected by differences in immune responses of the thrips vectors. Similarly, the silencing suppressor (NSs) might also have a role, as it has also been shown to influence virus accumulation in *F. occidentalis;* although its exact role is unknown, it likely suppresses the immune response^30^.

Behavior and fitness-related effects were not examined in this study but have been documented to influence transmission of plant viruses by their vectors. Infection of TSWV in male thrips increased feeding and the number of non-ingestion probes^48^, which are believed to be largely responsible for transmission because they leave cells intact^49^. Both infections in thrips and host plants significantly alter survival and development times of *F. occidentalis* and *F. fusca*, and the magnitude of these effects is influenced by host plant, virus isolate and temperature^3,4^. Effects of plant and vector infections on fitness of *T. tabaci* have not been reported, but may contribute to the lower transmission rates and higher titers observed in allopatric *T. tabaci-*TSWV isolate pairings. Although not formally studied, poor survival of *T. tabaci* on TSWV-infected plants was observed in some of the transmission experiments (Jacobson and Kennedy personal observation). Plant-virus interactions leading to changes in plant attractiveness to vectors are documented for plant viruses and their vectors^50^ but have not been studied in this pathosystem.

Previous work demonstrated that transmission efficiency of TSWV by *T. tabaci* isolines likely depends on genotypic interactions between virus isolates and *T. tabaci* isolines^17,21^. Our results extend these findings by demonstrating that variation in virus titer in adult thrips is not entirely responsible for the observed variation in transmission efficiency, and provide additional support for the importance of specific vector-virus interactions and co-evolutionary dynamics determining transmission efficiency within a single vector species. A better understanding of virus replication, movement, and accumulation in the vector is needed to determine whether transmission outcomes are influenced by more efficient localization or replication in the salivary glands. Understanding epidemiologically important patterns of variation in vector competence also requires a better understanding of the mechanisms underlying transmission, similarities of these mechanisms across sympatric vector species, and the influence of vector-imposed selection pressure on viral populations.

## Materials and Methods

### *T. tabaci* and TSWV: Collecting, culturing and transmission assays

*T. tabaci* individuals and TSWV isolates were subsamples of those tested by Jacobson and Kennedy^17^ in their characterization of differences in transmission efficiency among different pairings of *T. tabaci* isolines and TSWV isolates obtained from multiple locations and host plants in North Carolina. The clonal isolines of *T. tabaci* were established from thelytokous females (parthenogenetic reproduction - producing only female offspring) collected at each location to minimize genetic variation within each of the isolines. Transmission efficiency was then characterized by each of the isolate by isoline pairings. Details of collection, establishment of clonal thrips isolines, TSWV isolate establishment, transmission experiments, and results from transmission assays to characterize each TSWV isolate-isoline pairing are described in Jacobson and Kennedy^17^. Individual thrips were classified as transmitting or non-transmitting based on a DAS-ELISA test of the leaf discs on which viruliferous thrips were allowed to feed during the inoculation access period. Samples of transmitting thrips, non-transmitting thrips, and infected leaf tissue used for acquisition of TSWV from each isolate-isoline pairing were flash frozen in liquid nitrogen and stored at −80 °C until used in the experiments reported here. Twelve of the 89 isolate-isoline pairings for which transmission efficiencies were reported by Jacobson and Kennedy^17^ were included in the experiments reported here; they were representative of the range in transmission efficiencies observed among the 89 isolate-isoline pairings and included both sympatric and allopatric isolate-isoline pairings^17^ (Table 1). Three biological replicates of source plant tissue from each isolate-isoline pairing was included in this study. Up to five transmitting and non-transmitting thrips were selected per isolate-isoline pairing unless transmission rates were so low that five transmitting individuals were not observed in transmission experiments. The numbers of transmitting and non-transmitting thrips for each isolate-isoline pairing subjected to RT- qPCR are shown in Supplementary Table S1.

### Thrips and Leaf Disc RNA Extraction

Total RNA was extracted from 105 individual *T. tabaci* using TRIzol reagent (ThermoFisher Scientific, Waltham, Massachusetts) following the protocol used by Mason *et al*.^51^ with some modifications. For homogenization, each individual thrips was placed in a 1.5 ml microfuge tube, flash frozen in liquid N_2_, and then homogenized with a motorized micropestle (Kimble Chase, Vineland, NJ) followed by the addition of TRIzol. All incubation steps were at room temperature and all centrifugation steps were done at 16,000 x g. The pellets were air dried for 15 minutes, instead of vacuum concentrated, and resuspended in 8 µl (Diethyl pyrocarbonate; DEPC) treated water (Amresco, Solon, OH).

Total RNA was extracted from *Emilia sonchifolia* source leaf tissue (20 mg) with TRIzol using the manufacturers’ protocol. Homogenization was done using three Pyrex solid glass beads (3 mm; Corning, Corning, NY) in a 1.5 ml tube containing leaf tissue, flash frozen in liquid N_2_, and shaken for 20 seconds in a Silamat S6 mixer (Ivoclar Vivadent, Amherst, NY). Total RNA was resuspended in 50 µl of dH_2_O.

To ensure the integrity of flash frozen samples, nucleic acid concentrations of flash-frozen tissue were compared to fresh tissue samples for both *E. sonchifolia* and *T. tabaci*. Following total RNA extraction sample quality was confirmed by running the RNA on a gel to inspect quality as well as a NanoDrop 1000 (ThermoFisher Scientific, Waltham, MA) to confirm the quantity. Once the RNA extraction method was confirmed, it was used on the actual experiment samples and confirmed on the NanoDrop 1000.

### cDNA Synthesis

cDNA synthesis was required to convert total RNA to the DNA template required for RT-qPCR. First strand cDNA was synthesized from total RNA using ProtoScript II reverse transcriptase (New England Biolabs, Ipswich, MA). Thrips and leaf disc cDNA was synthesized using 2 µl of total RNA in a 20 µl reaction primed with 2 µl of random hexamers (60 µM; Invitrogen, Carlsbad, CA). The primers, 1 µl dNTP mix (10 mM), total RNA and dH_2_O were combined to a final volume of 12 µl, heated at 70 °C for 5 minutes and chilled immediately on ice before the remaining reagents were added (ProtoScript reaction buffer, DTT (10 mM final), Murine RNase inhibitor (2U/µl final) and ProtoScript II reverse transcriptase (20U/µl final). The prep was then incubated at 25 °C for 5 minutes followed by incubation at 42 °C for 1 hour. The enzyme was inactivated at 80 °C for 5 minutes and stored at −20 °C until used for RT-qPCR.

### Quantitative Real-Time PCR (RT-qPCR) Primer design

There are no published RT-qPCR primers nor was there a complete genome sequence for *T. tabaci*. Previously reported *F. occidentalis* actin primers as well as other internal control genes were assayed as possible internal control genes for *T. tabaci*^19,52,53^ (Supplementary Table S2). Primers were also designed and validated for the commonly used internal control, Elongation factor one alpha (EF1A), from an alignment of 7 published partial *T. tabaci* EF1A sequences (Genbank accession numbers: KM582809, AB894111, AB277263, AB277262, AB894109, AB894108, AB277575) (Supplementary Table S3).

Because *E. sonchifolia* does not have published RT-qPCR primers or a complete genome sequence, and previously published RT-qPCR primers developed from the highly conserved common plant genes actin, tRNA and profilin^19^. An alignment of 3 partial *E. sonchifolia* sequences for the 5.8 *S* rRNA gene and internal transcribed spacer (ITS) were also utilized to design additional primer pairs (accession numbers: JF733772, KU696022, MF440623) (Supplementary Table S4).

TSWV quantification was validated using a number of published RT-qPCR primers specific to the nucleocapsid (N) gene of the small (S) RNA segment^16,19,54,55^. Primers were also designed spanning the L RNA segment using an alignment of 5 published sequences (Genbank accession numbers: NC_002052, JN664254, HM581940, HM581934, JF960237) as another possible measurement of TSWV transcript levels in both thrips and infected source leaf tissue (Supplementary Table S5).

### RT-qPCR

Viral titer was quantified using the primer efficiency method, in which the target gene expression is quantified relative to the expression of an internal control while accounting for primer efficiency^56,57^. Primer efficiency values (E) were calculated by 10^(−1/slope)^ where the slope of the standard curve was obtained by plotting the concentration of five, five-fold dilutions of a single cDNA reaction made from thrips with a high TSWV titer against their Ct values. TSWV transcript levels were normalized to the internal controls by the inverse ratio of Pfaffl^57^: E_internal control_^Ct (internal control)^ /E_L_^Ct (L)^.

RT-qPCR was performed on a QuantStudio 6 Flex system (Applied Biosystems, Foster City, CA) with a 96-well fast block. iTaq Universal SYBR Green Supermix, SsoAdvanced SYBR Green Supermix, and SsoFast EvaGreen Supermix (all Bio-Rad, Hercules, CA) were compared for their ability to detect amplification of DNA. SsoAdvanced SYBR Green Supermix was chosen because it had the most reproducible results. All samples were run in triplicate for each primer pair to control for pipetting errors, along with no-template controls (Table 2). Reactions (20 µl) were performed in 0.1 ml 96-well plates using the manufacturer’s recommended protocol: initial denaturation at 95 °C for 30 seconds, 40 cycles of denaturation at 95 °C for 15 seconds and annealing/extension at 60 °C for 30 seconds, followed by a single melt curve stage of 95 °C for 15 seconds, 60 °C for 60 seconds, and 95 °C for 15 seconds. The number of denaturation/annealing/extension cycles was reduced to 30 for the source leaf tissue due to the higher initial RNA concentrations obtained.

### Statistical analysis

Virus titers were expressed as the normalized values for the relative TSWV transcript levels in the leaf tissue used as the virus source for acquisition by the thrips or in individual thrips. Titers in the leaf discs used as virus sources were log transformed (base 10) and subjected to one-way ANOVA to test for differences among isolate-isoline pairings.

Further analyses examining the relationships between virus titer in individual thrips, virus isolate, and thrips isoline were conducted using the GLIMMIX procedure of the SAS system version 9.4 (SAS Institute, Cary, NC). Normalized values for the relative TSWV transcript levels were analyzed using a generalized linear mixed model with an assumed lognormal response distribution. An initial analysis tested a model in which the independent variables were virus isolate, isoline and their interaction. This relationship was examined further in a second model in which the interaction term was replaced with the variable “collection location,” in which virus isolate and isoline pairings were grouped as sympatric or allopatric. Similar analyses were conducted to test the association between virus titer in thrips and probability of transmission. In these analyses, transmission was treated as a binary variable with individual thrips in our subsamples classified as transmitting or non-transmitting. We could not directly test for associations between titers and transmission efficiencies of specific isolates-isoline pairings because the numbers of transmitting thrips were too low for some pairings to ensure inclusion of a sufficient number of transmitting thrips in a random sample of available thrips. Therefore, the thrips samples from each isolate-isoline pairing subjected to RT-qPCR were chosen to ensure adequate representation of transmitting and non-transmitting thrips.

## Supplementary information

Supplementary Information.

## Data Availability

The datasets generated or analyzed and not included in the manuscript (and its Supplemental Information files) are available from the corresponding author on reasonable request.
