# Supplementary material for: Relationships of virus titers and transmission rates among sympatric and allopatric virus isolates and thrips vectors support local adaptation
Source: Sci Rep. 2020 May 6;10:7649. doi: 10.1038/s41598-020-64507-1 (PMC7203134; doi:10.1038/s41598-020-64507-1)
Supplement: Supplementary file 1 — Supplementary Information. [file 41598_2020_64507_MOESM1_ESM.pdf]

Relationships of virus titers and transmission rates among sympatric and allopatric virus isolates and thrips vectors support local adaptation

Jessica A. Linak[1], Alana L. Jacobson[2], Tim L. Sit[1], and George G. Kennedy[1]

[1]Department of Entomology and Plant Pathology, North Carolina State University, Raleigh, NC 27695-7630; [2]Department of Entomology and Plant Pathology, 301 Funchess Hall, Auburn University, Auburn, AL, 36849, USA

Contact Author: Alana L Jacobson alj0043@auburn.edu

Table S1. The tomato spotted wilt virus (TSWV) isolate-*Thrips tabaci isoline*-pairings included in this study, and the number of adult *T. tabaci* in each isoline that transmitted or did not transmit each TSWV isolate. Thrips and leaf tissue used in study are the same reported in Jacobson and Kennedy (2013) and were stored at -80°C until used in this study.

		Number of thrips		
TSWV Isolate	Thrips Isoline	Transmitting	Nontransmitting	Total
AM1	IPOC1*	5	5	10
AM1	Kin1	5	5	10
AM1	SH2	4	5	9
AM1	SH72	4	5	9
SH3	IPOC1	2	6	8
SH3	Kin1	1	4	5
SH3	SH2*	5	5	10
SR3-3	IPOC1*	3	5	8
SR3-3	Kin1	2	5	7
SR3-3	SH2	4	5	9
SR3-3	SH72	3	6	9
SHP	SH2*	6	5	11
Totals		44	61	105

Table S2. *Frankliniella occidentalis* primers tested as possible internal control genes for *Thrips tabaci* qPCR experiments.

Gene	Primer Name	Primer Sequence (5'-3')	Reference
β-actin	WFT-RNA-25F	F: GGTATCGTCCTGGACTCTGGTG	*20*
	WFT-RNA-26F	F: GTATCGTCCTGGACTCTGGTGA	*20*
	WFT-RNA-93R-C	R: GGGGAAGGGCGTAACTTCA	*20*
	WFT-RNA-94R-B	R: GGGGAAGGGCGTAACCTTC	*20*
	WFT-RNA-93R-D	R: GGGGAAGGGCGTAACCTTCAT	*20*
	WFT-RNA-92R	R: GGGGAAGGGCGTAACCTTCATAG	*20*
	Actin F	CCTCATCCCTAGTTGTCTTGTG	*57*
	Actin R	TTCTCGCTCAGCTGTAATTGT	
NADH-ubiquinoneoxidoreductase	NADH	F: AGCTACTAAACCGCCTCATAAA	*57*
		R: GGTGGTTATGGTATTTATCGTTTGT	
α-tubulin	Tubulin	F: GTGGACAACGAAGCCATCTA	*57*
		R: CGGTTCAGGTTGGTGTAGG	
heat shock protein 90	HSP90	F: CTCGCAACCAGGACGATATTAG	*57*
		R: CTGACCCTCCACAGAGAAATG	
heat shock protein 70	HSP70	F: GTCACCGTACCCGCATATTT	*57*
		R: GCAGTGGGCTCGTTGATAATA	
heat shock protein 60	HSP60	F: CTGGACTGTAAGCGTGCTATAA	*57*
		R: GGCACGATGAACACCTATGA	
vacuolar type H+ -ATPase	ATPase	F: TACCAAATGGGACTCCAATACC	*57*
		R: GTAAGTAAGAGGTGGCCAGATAC	
ribosomal protein l32	RPL32	F: CTGGCGTAAACCTAAGGGTATT	*57*
		R: GTCTTGGCATTGCTTCCATAAC	
		F: CAACATCGGTTATGGAAGCA	*58*
		R: ACAGCGTGGGCAATTTCAGC	
28*S* ribosomal RNA	28*S*	F: GGGTGGTAAACTCCATCTAAGG	*57*
		R: CACGTACTCTTGAACTCTCTCTTC	
18*S* ribosomal RNA	18*S*	F: CTGCGGAAATACTGGAGCTAATA	57
		R: AAGTAGACGATGGCCGAAAC	
		F: AACACGGGAAACCTCACCA	*58*
		R: CAGACAAATCGCTCCACCAA	
elongation factor 1 α	EF1A	F: AAGGAACTGCGTCGTGGATA	*57*
		R: AGGGTGGTTCAGGACAATGA	

EF-1 F	TCAAGGAACTGCGTCGTGGAT	*58*
EF-1 R	ACAGGGGTGTAGCCGTTAGAG	

Table S3. *Thrips tabaci* EF1A primers designed from existing Genbank sequences and tested as possible internal control genes for qPCR.

Primer Name	Sequence
EF1A_315F	CGACAACGTTGGTTTCAACATC
EF1A_448R	GTAGCCGTTGGAGATCTGGCCAGG
EF1A_372F	CGTCGCTGGTGACTCCAAG
EF1A_478R	GGCGGTGTGGCAATCCAGCAC
**EF1A_346F**	**CGTCAAGGAACTTCGTCGTG**
EF1A_322F	GTCGGCTTCAACATCAAGAACG
EF1A_414F	CGACTTCACCGCACAGGT
EF1A_481R	AGCGGTGTGGCAATCCAG
EF1A_431R	ACCTGTGCGGTGAAGTCG
**EF1A_456R**	**CACAGGGGTGTATCCGTTG**

Table S4. *Emilia sonchifolia* internal control primers designed from existing Genbank sequences and tested as possible internal control genes for qPCR.

**Gene**	**Primer Name**	**Primer Sequence**	**Reference**
Sunflower profilin	Heli-all-nes_f2	CGTCAATACTTGTTAATATTATTAAGAATTA	59
	Heli-all-r1	ATAGCTTGGCCCGTTTTCTT	59
Plant tRNA	Plant_nes-2-f	ATTGAGCCTTGGTATGGAAACCT	59
	Plant_nes-2-r	GGATTTGGCTCAGGATTGCC	59
Plant actin	Act-f	CAAGCAGCATGAAGATCAAGGT	59
	Act-r	CACATCTGTTGGAAAGTGCTGAG	59
***E. sonchifiolia* 5.8S rRNA**	**Emilia_5.8S_rRNAF**	**GTGTGAATTGCAGAATCCCGT**	
	**Emilia_5.8S_rRNAR**	**CATGTGACGCCCAGGCA**	
E. sonchifolia ITS	Emilia_ITS2_F	GTCACCTCCCAACACACCT	
	Emilia_ITS2_R	GCTTTTCGACCACCACTAATC	

Table S5. *Tomato spotted wilt virus* target gene primers from existing literature, and newly designed primers tested as targets for qPCR reactions.

Gene	Primer Name	Primer Sequence	Reference
Nucleocapsid	TSWV-CP-17F	F: CTCTTGATGATGCAAAGTCTGTGA	20
	TSWV-CP-100R	R: TCTCAAAGCTATCAACTGAAGCAATAA	
	TSWV N F	F: GCTTCCCACCCTTTGATTC	17
	TSWV N R	R: ATAGCCAAGACAACACTGATC	
	TSW.1	F: TCTGGTAGCATTCAACTTCAA	21
	TSW.2	R: GTTTCACTGTAATGTTCCATA	
	TSWV-1 For	F: AGACAGGATTGGAGCCACTGACAT	22
	TSWV-1 Rev	R: TCCCAGTTTCCTCAACAAGCCTGA	
L RNA	TSWVL_746F	F: GGATGCCAATCACTGTTACTAG	
	TSWVL_884R	R: CACCACTGTGGGTGTGG	
	TSWVL_2696F	F: CTGTCATGATAGGAACTGTGAC	
	TSWVL_2877R	R: GAGATCTTCCATTCTGAAC	
	TSWVL_4382F	F: GCATGAAYTGGTTRCAAGGC	
	TSWVL_4493R	R: CAGAGTGCACAATCCATCTAG	
	TSWVL_4656R	R: CRGATGAAGAAGCATAACTC	
	TSWVL_4890F	F: CTATTCAATGCTTCCTGGTG	
	TSWVL_4966R	R: CTCTAAGGGTGTTTGTACC	
	TSWVL_6394F	F: CTGGACACTGCTGTATACATATC	
	TSWVL_6585R	R: GTGTTCAATTCACTGTTATGTG	
	TSWVL_6766F	F: GGGATCATGACAAGAAGCTG	
	TSWVL_6970R	R: GCAYGCATCRCCTGGTTC	
	TSWV_7712F	F: CATGCCACAACAATGACTC	
	TSWVL_7919R	R: GRTTCTCTCCAGATATAACAAACC	
	TSWVL_7993F	F: GTGTTGAGGCTAGATGAGG	
	TSWVL_8192R	R: CTRCCCARTCTGTCAGTTG	

Table S6. Individual *Thrips tabaci* Ct values, Standard deviations, Delta Ct (normalized virus titer values), and log10 transformed Delta Ct values. Shaded rows = transmitting thrips; Non-shaded rows = non-transmitting thrips.

			Internal Control		Viral L RNA					
Virus Isolate	Thrips Isoline	Sympatry	EF1A Ct	EF1A SD	TSWV L Ct	TSWV L SD	Delta Ct SD	Delta Ct SE	Delta Ct Mean	Log10 Delta Ct
AM1	IPOC1-1	Sympatric	19.514	0.144	21	0.163	1.149	1.083	0.204	-0.69
AM1	IPOC1-2	Sympatric	21.326	0.043	23.42	0.169	1.12	1.068	0.13	-0.887
AM1	IPOC1-3	Sympatric	21.548	0.088	23.514	0.15	1.119	1.067	0.14	-0.854
AM1	IPOC1-4	Sympatric	22.911	0.035	35.675	0.579	1.46	1.244	0	-3.932
AM1	IPOC1-5	Sympatric	29.619	0.334	38.096	0	0	0	0.002	-2.81
AM1	IPOC1-6	Sympatric	20.791	0.018	36.156	2.134	4.024	2.676	0	-4.64
AM1	IPOC1-7	Sympatric	20.718	0.008	36.444	1.007	1.929	1.461	0	-4.741
AM1	IPOC1-8	Sympatric	18.416	0.185	21.763	0.152	1.112	1.063	0.055	-1.26
AM1	IPOC1-9	Sympatric	16.76	0.206	32.358	0.555	1.468	1.248	0	-4.65
AM1	IPOC1-10	Sympatric	20.896	0.076	36.934	0.039	1.055	1.033	0	-4.832
AM1	Kin1-1	Allopatric	21.496	0.016	24.79	0.48	1.367	1.198	0.059	-1.23
AM1	Kin1-2	Allopatric	17.577	0.236	21.513	0.163	1.199	1.11	0.044	-1.357
AM1	Kin1-3	Allopatric	17.759	0.072	21.619	0.068	1.065	1.037	0.046	-1.338
AM1	Kin1-4	Allopatric	18.852	0.055	22.502	0.248	1.18	1.1	0.051	-1.294
AM1	Kin1-5	Allopatric	25.354	0.082	27.043	0.052	1.063	1.036	0.149	-0.828
AM1	Kin1-6	Allopatric	19.891	0.023	22.455	0.229	1.162	1.091	0.1	-1.001
AM1	Kin1-7	Allopatric	19.185	0.086	22.337	0.092	1.084	1.048	0.07	-1.158
AM1	Kin1-8	Allopatric	19.591	0.376	24.167	0.178	1.297	1.162	0.027	-1.566
AM1	Kin1-9	Allopatric	27.389	0.097	31.6	0.087	1.086	1.049	0.027	-1.571
AM1	Kin1-10	Allopatric	20.958	0.01	34.622	0.517	1.401	1.215	0	-4.16
AM1	SH2-1	Allopatric	20.621	0.239	24.111	0.173	1.205	1.113	0.053	-1.273
AM1	SH2-2	Allopatric	21.581	0.091	21.043	0.258	1.194	1.108	0.716	-0.145
AM1	SH2-3	Allopatric	19.993	0.061	37.157	0	0	0	0	-5.138
AM1	SH2-4	Allopatric	20.3	0.058	23.036	0.088	1.07	1.04	0.088	-1.055
AM1	SH2-5	Allopatric	19.383	0.078	22.026	0.065	1.066	1.038	0.096	-1.016
AM1	SH2-6	Allopatric	20.695	0.011	36.177	0	0	0	0	-4.671
AM1	SH2-7	Allopatric	17.214	0.022	33.781	0.605	1.484	1.322	0	-4.931
AM1	SH2-8	Allopatric	18.25	0.074	35.38	0.053	1.059	1.037	0	-5.105
AM1	SH2-9	Allopatric	18.14	0.042	34.042	0.642	1.521	1.345	0	-4.755
AM1	SH72-1	Allopatric	19.842	0.193	28.218	0.076	1.139	1.078	0.002	-2.646

AM1	SH72-2	Allopatric	20.025	0.072	32.05	0.201	1.148	1.083	0	-3.683
AM1	SH72-3	Allopatric	17.976	0.093	36.208	0.766	1.653	1.337	0	-5.413
AM1	SH72-4	Allopatric	17.881	0.359	35.354	0	0	0	0	-5.197
AM1	SH72-5	Allopatric	19.281	0.188	27.362	0.399	1.33	1.179	0.003	-2.555
AM1	SH72-6	Allopatric	19.169	0.403	31.074	0.596	1.588	1.306	0	-3.637
AM1	SH72-7	Allopatric	17.761	0.082	26.299	0.046	1.061	1.035	0.002	-2.664
AM1	SH72-8	Allopatric	17.924	0.028	26.41	0.056	1.042	1.024	0.002	-2.651
AM1	SH72-9	Allopatric	19.609	0.021	37.413	0.838	1.727	1.371	0	-5.314
SH3	IPOC1-1	Allopatric	19.025	0.035	20.516	0.022	1.026	1.015	0.207	-0.685
SH3	IPOC1-2	Allopatric	23.874	0.075	24.273	0.129	1.101	1.057	0.361	-0.442
SH3	IPOC1-3	Allopatric	18.187	0.085	23.437	0.184	1.14	1.079	0.018	-1.738
SH3	IPOC1-4	Allopatric	25.445	0.024	26.355	0.046	1.034	1.02	0.246	-0.609
SH3	IPOC1-5	Allopatric	23.22	0.137	35.325	2.007	3.713	2.527	0	-3.749
SH3	IPOC1-6	Allopatric	19.814	0.004	22.984	0.011	1.007	1.004	0.067	-1.171
SH3	IPOC1-7	Allopatric	17.43	0.14	32.023	0.18	1.157	1.088	0	-4.375
SH3	IPOC1-8	Allopatric	17.843	0.025	33.151	0.196	1.138	1.077	0	-4.583
SH3	Kin1-1	Allopatric	25.468	0.328	35.393	0	0	0	0.001	-3.163
SH3	Kin1-2	Allopatric	19.288	0.278	33.775	0.457	1.412	1.22	0	-4.37
SH3	Kin1-3	Allopatric	19.801	0.163	33.902	0.345	1.28	1.153	0	-4.268
SH3	Kin1-4	Allopatric	26.38	0.293	36.954	0	0	0	0	-3.359
SH3	Kin1-5	Allopatric	17.613	0.266	30.873	0.19	1.229	1.126	0	-4
SH3	SH2-1	Sympatric	20.39	0.017	36.044	0.685	1.564	1.372	0	-4.716
SH3	SH2-2	Sympatric	17.822	0.047	36.781	0.136	1.098	1.067	0	-5.617
SH3	SH2-3	Sympatric	16.814	0.01	35.264	0.904	1.803	1.405	0	-5.459
SH3	SH2-4	Sympatric	17.736	0.033	35.362	0.679	1.558	1.292	0	-5.238
SH3	SH2-5	Sympatric	18.826	0.014	35.478	1.369	2.443	1.675	0	-4.977
SH3	SH2-6	Sympatric	18.207	0.08	36.599	0	0	0	0	-5.462
SH3	SH2-7	Sympatric	20.203	0.014	35.935	0.752	1.634	1.328	0	-4.736
SH3	SH2-8	Sympatric	16.318	0.04	36.075	1.116	2.071	1.673	0	-5.822
SH3	SH2-9	Sympatric	17.263	0.06	34.887	0	0	0	0	-5.231
SH3	SH2-10	Sympatric	17.353	0.03	35.854	0.983	1.899	1.448	0	-5.481
SR3-3	IPOC1-1	Sympatric	23.539	0.01	25.903	0.023	1.017	1.01	0.101	-0.994
SR3-3	IPOC1-2	Sympatric	36.016	0	36.207	0	0	0	0.281	-0.551
SR3-3	IPOC1-3	Sympatric	19.802	0.006	22.218	0.073	1.049	1.028	0.11	-0.957
SR3-3	IPOC1-4	Sympatric	20.321	0.068	34.678	1.007	1.932	1.593	0	-4.348

SR3-3	IPOC1-5	Sympatric	19.597	0.314	37.249	0	0	0	0	-5.271
SR3-3	IPOC1-6	Sympatric	21.626	0.058	36.914	0.231	1.167	1.114	0	-4.629
SR3-3	IPOC1-7	Sympatric	21.351	0.067	24.651	0.035	1.049	1.028	0.059	-1.229
SR3-3	IPOC1-8	Sympatric	19.673	0.026	21.937	0.01	1.018	1.01	0.122	-0.913
SR3-3	Kin1-1	Allopatric	23.856	0.063	25.507	0.041	1.049	1.028	0.16	-0.797
SR3-3	Kin1-2	Allopatric	18.779	0.052	22.546	0.02	1.036	1.02	0.047	-1.326
SR3-3	Kin1-3	Allopatric	22.878	0.134	35.831	0.266	1.212	1.141	0	-3.985
SR3-3	Kin1-4	Allopatric	21.337	0.102	35.025	0.483	1.379	1.204	0	-4.172
SR3-3	Kin1-5	Allopatric	19.501	0.124	34.377	0.608	1.497	1.263	0	-4.483
SR3-3	Kin1-6	Allopatric	18.88	0.024	31.279	0.035	1.028	1.016	0	-3.773
SR3-3	Kin1-7	Allopatric	23.009	0.056	35.132	0	0	0	0	-3.752
SR3-3	SH2-1	Allopatric	20.869	0.116	23.071	0.096	1.1	1.057	0.123	-0.912
SR3-3	SH2-2	Allopatric	23.639	0.114	36.64	0	0	0	0	-4.009
SR3-3	SH2-3	Allopatric	20.394	0.074	22.957	0.028	1.05	1.029	0.098	-1.007
SR3-3	SH2-4	Allopatric	21.708	0.092	24.851	0.055	1.07	1.04	0.065	-1.19
SR3-3	SH2-5	Allopatric	20.952	0.318	21.864	0.093	1.23	1.127	0.284	-0.547
SR3-3	SH2-6	Allopatric	19.744	0.031	22.414	0.007	1.02	1.011	0.094	-1.029
SR3-3	SH2-7	Allopatric	20.203	0.113	36.206	0	0	0	0	-4.812
SR3-3	SH2-8	Allopatric	34.867	0.928	32.584	1.04	2.435	1.672	1.466	0.166
SR3-3	SH2-9	Allopatric	20.103	0.015	22.796	0.033	1.024	1.014	0.091	-1.04
SR3-3	SH72-1	Allopatric	38.784	0.07	35.699	1.326	2.378	1.649	2.184	0.339
SR3-3	SH72-2	Allopatric	20.577	0.034	23.067	0.032	1.03	1.017	0.103	-0.989
SR3-3	SH72-3	Allopatric	19.537	0.014	26.011	0.027	1.15	1.084	0.008	-2.073
SR3-3	SH72-4	Allopatric	20.278	0.215	26.609	0.063	1.294	1.161	0.013	-1.892
SR3-3	SH72-5	Allopatric	19.981	0.047	25.004	0.18	1.129	1.072	0.02	-1.698
SR3-3	SH72-6	Allopatric	25.792	0.067	0	0	0	0	0	0
SR3-3	SH72-7	Allopatric	22.153	0.11	35.986	0	0	0	0	-4.225
SR3-3	SH72-8	Allopatric	20.765	0.089	35.481	0.025	1.059	1.034	0	-4.456
SR3-3	SH72-9	Allopatric	29.948	0.166	35.95	0.652	1.549	1.358	0.008	-2.113
SHP	SH2-1	Sympatric	18.822	0.108	21.629	0.107	1.102	1.057	0.088	-1.055
SHP	SH2-2	Sympatric	18.391	0.046	22.988	1.456	2.587	1.731	0.028	-1.556
SHP	SH2-3	Sympatric	20.036	0.164	22.595	0.166	1.16	1.09	0.1	-1.001
SHP	SH2-4	Sympatric	19.764	0.046	22.956	0.082	1.063	1.036	0.067	-1.177
SHP	SH2-5	Sympatric	23.045	0.074	24.37	0.077	1.071	1.04	0.203	-0.693
SHP	SH2-6	Sympatric	26.503	0.065	28.727	0.089	1.073	1.042	0.101	-0.996

SHP	SH2-7	Sympatric	18.23	0.381	24.342	0.04	1.269	1.147	0.01	-1.983
SHP	SH2-8	Sympatric	27.938	0.198	36.227	0	0	0	0.002	-2.734
SHP	SH2-9	Sympatric	18.354	0.078	33.948	0.915	1.82	1.413	0	-4.671
SHP	SH2-10	Sympatric	19.939	0.078	21.807	0.087	1.077	1.044	0.157	-0.804
SHP	SH2-11	Sympatric	18.55	0.042	23.214	0.062	1.049	1.028	0.026	-1.577

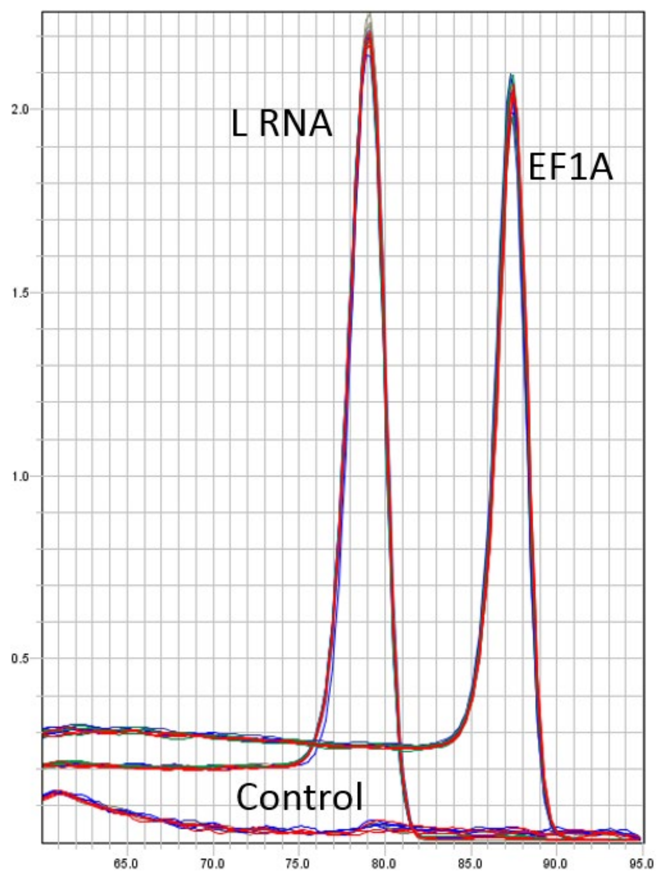

Figure S1. *Thrips tabaci* internal control and L RNA melt curves. The thrips internal control (EF1A) primers and TSWV target (L RNA) are shown for multiple samples with sharp single peaks indicating high specificity, amplification, and one product for each primer pair. Negative controls with no template controls are also shown to indicate no contamination or primer dimers.

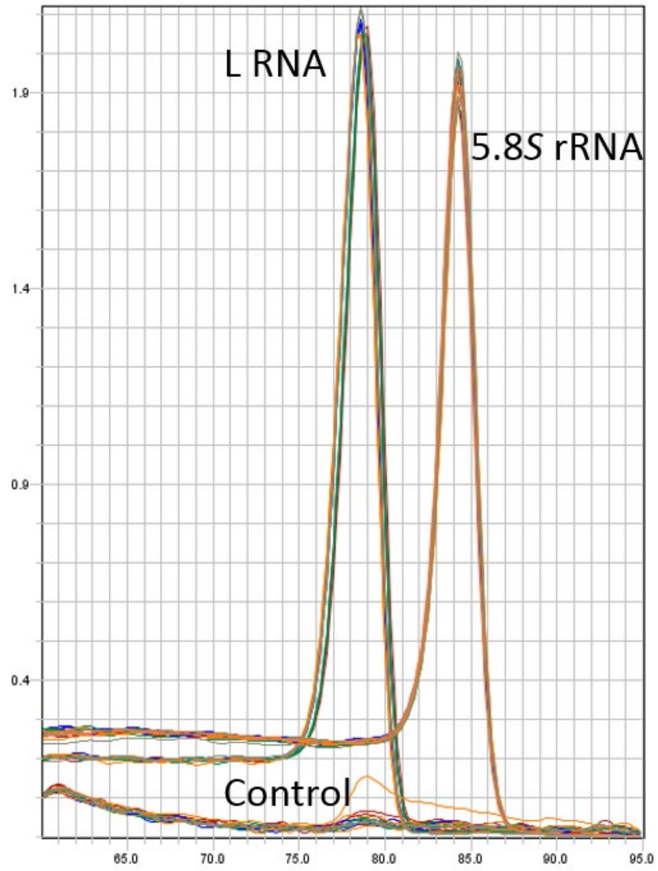

Figure S2. *Emilia sonchifolia* internal control and L RNA melt curves. The leaf tissue internal control (5.8*S* rRNA) primers and TSWV target (L RNA) are shown for multiple samples with sharp single peaks indicating high specificity, amplification, and one product for each primer pair. Negative controls with no template are also shown to indicate no contamination or primer dimers.

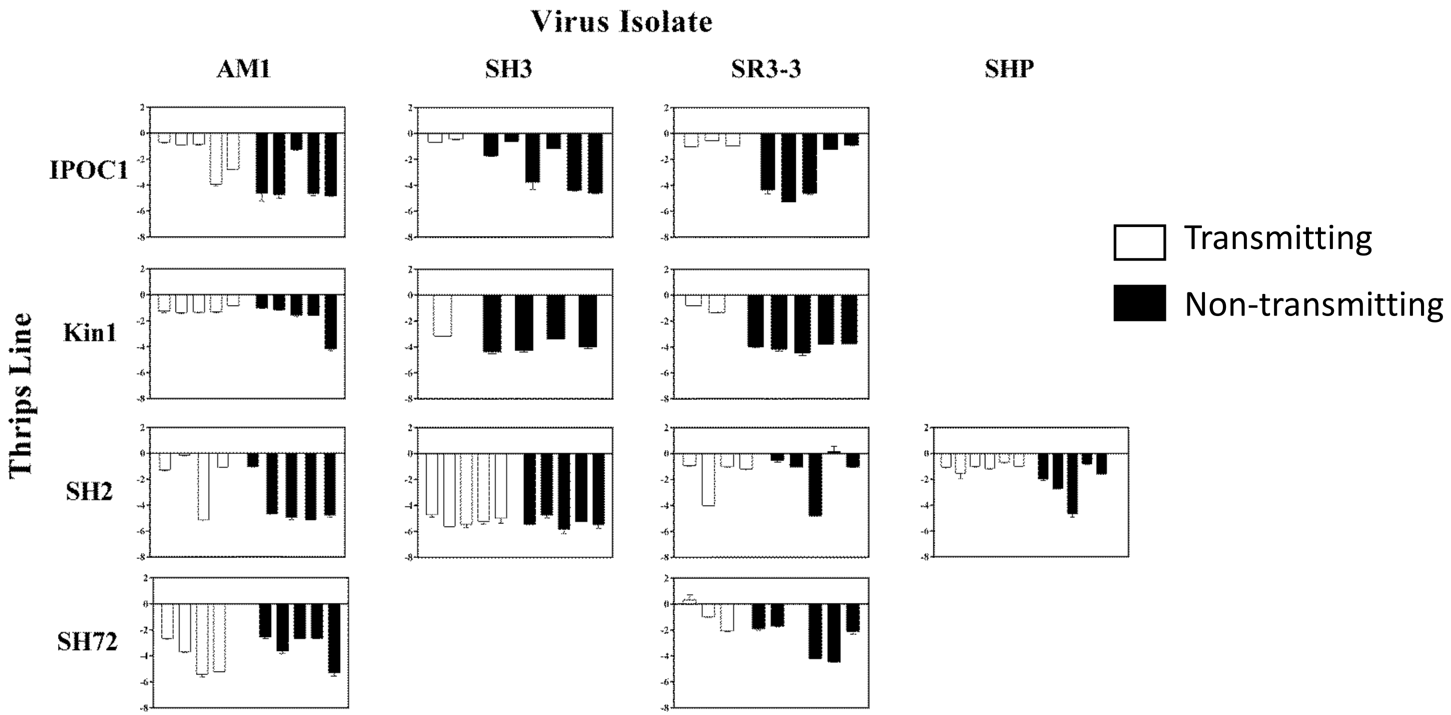

Figure S3. Normalized $\log_{10}$ transformed virus titer for each individual *Thrips tabaci* for all thrips tested. Means are presented for each single *T. tabaci* tested for TSWV titer and shown for all virus isolates and thrips isofemale lines separately. Transmitting individuals are in white solid bars and non-transmitting individuals are in black solid bars.
